# Recurrent Ascites Beyond the Usual Suspects: Uncovering an Overlooked Culprit

**DOI:** 10.1155/crhe/9961539

**Published:** 2025-05-24

**Authors:** Binoy Desai, Harjit Singh, Alessandra Martorella, Bryce Kunkle, Amol S. Rangnekar

**Affiliations:** ^1^Mount Sinai Health System, New York, New York, USA; ^2^MedStar Georgetown University Hospital, Washington, District of Columbia, USA

## Abstract

Hepatic amyloidosis is a rare condition that leads to progressive liver dysfunction. Diagnosis is often challenging since clinical presentation may be highly varied and is dependent upon the extent of liver involvement, underlying etiology of amyloid deposition, as well as concomitant extrahepatic manifestations. Ascites, although uncommon, can be a presenting feature of hepatic amyloidosis and pose diagnostic challenges as it can occur in a variety of liver and nonliver-related conditions. Herein, we present a case of hepatic amyloidosis in a patient with recurrent ascites, emphasizing the importance of considering this condition in the differential diagnosis of individuals presenting with unexplained ascites.

## 1. Introduction

Light chain–associated (AL) or primary amyloidosis is a rare disease characterized by the abnormal accumulation of insoluble misfolded protein fibrils in various organs and tissues throughout the body. Hepatic amyloidosis is a rare manifestation of systemic amyloidosis distinguished by the deposition of amyloid fibrils within the liver parenchyma, and clinical manifestation is often highly variable [1]. Hepatic involvement is typically seen in up to 90% of patients [2]. However, the clinical symptoms are usually mild and include hepatomegaly, an elevated alkaline phosphatase, and weakness. Although very rare, without appropriate management of hepatic amyloidosis, it can lead to worsening liver function and complications over time such as portal hypertension, jaundice, ascites, coagulopathy, edema, or liver failure [2, 3]. Ascites as a presenting feature of hepatic amyloidosis is relatively rare and can mimic other etiologies of liver disease, posing diagnostic challenges for clinicians. In this report, we present a rare case of hepatic amyloidosis in a patient with recurrent and unexplained ascites.

## 2. Case Report

A 70-year-old male with a past medical history of end-stage renal disease (ESRD) secondary to AL amyloidosis presented with fatigue, dizziness, and abdominal distension of several months. He had been treated with an autologous bone marrow transplant and melphalan for amyloidosis-induced ESRD previously. His amyloidosis was previously confined to the kidneys only.

Initial laboratory findings revealed an acute anemia with hemoglobin of 7.4 g/dL and thrombocytopenia of 70 thous/μL. Coagulation studies and liver function tests were within normal limits: INR of 1.2, alanine transaminase of 9 IU/L, aspartate transaminase of 13 IU/L, alkaline phosphatase of 135 IU/L, albumin of 3.7 g/dL, and total bilirubin of 0.73 mg/dL. Triple-phase computed tomography (CT) imaging of the abdomen revealed large-volume ascites and evidence of capsular hematoma on the posterior aspect of the spleen without evidence of cirrhosis, portal vein thrombosis, or hepatocellular carcinoma. Given these findings, the patient underwent paracentesis which showed evidence of hemorrhagic ascites with 3,835,000 RBCs/mm^3^. There were 2274 nucleated cells/mm^3^ with 51% segmented neutrophils, 26% monocytes/macrophages, 14% mesothelial cells, and 9% lymphocytes. The total protein concentration in the ascitic fluid was elevated at 4.2 g/dL. The ascitic albumin was 0.28 g/dL, resulting in a serum-to-ascites albumin gradient (SAAG) of 3.4 g/dL.

**Figure 1:**
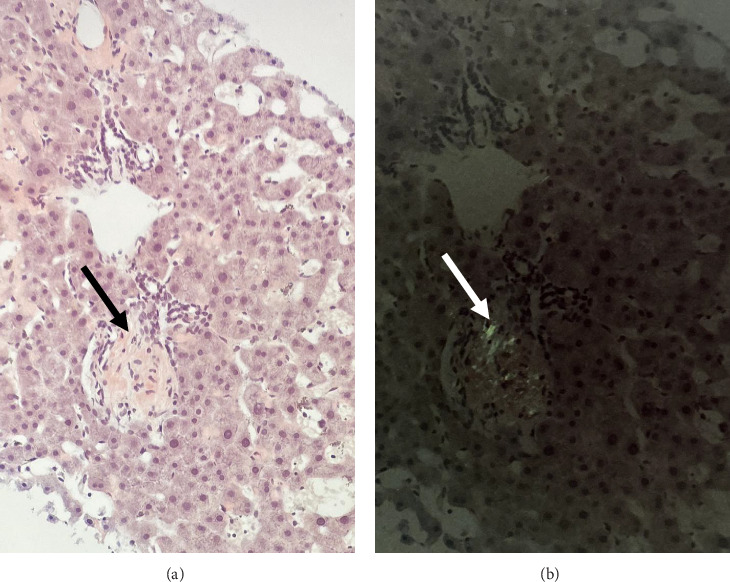
(a) H&E stain, 20x. Section of hepatic parenchyma showing accumulation of amorphous material around portal tract (arrow). (b) Congo red stain, 20x. Polarized section showing apple-green birefringence concordant with amyloid deposition (arrow).

To further evaluate the etiology of his ascites, a full liver and cardiac workup was pursued. His echocardiogram showed normal ejection fraction with mild diastolic dysfunction. Liver serologies to evaluate for other causes of liver disease including autoimmune, viral, metabolic, storage disorders, and genetic disorders were all negative. A transjugular liver biopsy was pursued and revealed an elevated hepatic venous pressure gradient (HVPG) of 11 mmHg, sinusoidal dilation, and evidence of venous outflow impediment with positive Congo red staining consistent with hepatic amyloidosis (Figure 1). Although there was a clinical concern for possible sinusoidal obstruction syndrome given his prior melphalan exposure, there was no histologic evidence of this condition.

Based on the clinical, imaging, and pathological findings, the patient was diagnosed with hepatic amyloidosis. His symptoms were managed with recurrent paracenteses. Unfortunately, he developed hemorrhagic shock and passed away due to associated complications.

## 3. Discussion

Our case demonstrates that hepatic amyloidosis is an uncommon cause of ascites, but one that should be considered in the differential diagnosis of recurrent unexplained ascites. The presence of ascites in hepatic amyloidosis is rare and has been seldom reported in the literature. Most patients do not develop symptoms from hepatic amyloidosis. Histological studies of the liver in patients with AL amyloidosis have revealed a high incidence of hepatic infiltration, but epidemiological data suggest that only up to 25% of patients will present with symptoms from hepatic involvement [4, 5].

Hepatic amyloid compresses and replaces normal liver tissue leading to organ dysfunction. Portal hypertension, a rare complication of hepatic amyloidosis, is typically related to the degree of hepatic infiltration and develops due to the reduction of the vascular space of hepatic sinusoids by large perisinusoidal amyloid deposits [1, 4]. In this patient, both the elevated HVPG and SAAG were indicative of portal hypertension. Furthermore, the ascitic fluid demonstrated a high total protein concentration, consistent with a protein-rich exudate associated with underlying liver dysfunction. Portal hypertension is a poor prognostic indicator and often leads to subsequent liver failure [3, 6].

Diagnosis of hepatic amyloidosis is complex and challenging. The most common presenting symptoms include hepatomegaly and an elevated alkaline phosphatase, which are nonspecific and often not helpful in diagnosis [4]. In addition, there are no specific radiologic findings either, but there may be evidence of diffuse or focally decreased parenchymal attenuation, irregular calcifications, or triangular-shaped hepatomegaly on CT scan [4, 7]. A liver biopsy is necessary for the definitive diagnosis of hepatic amyloidosis, and subsequent histopathologic examination reveals characteristic apple-green birefringence with Congo red staining of the amyloid fibrils deposited within the liver tissue [4].

Overall, hepatic amyloidosis portends a poor prognosis. Most patients survive less than a year from initial diagnosis, and one study showed a median survival time of 8.5 months in 98 patients with biopsy-proven hepatic amyloidosis [5]. Treatment options are limited and primarily focus on managing symptoms and addressing associated complications. For patients with AL amyloidosis, chemotherapy and stem cell transplant are the mainstay treatments. Patients are usually treated with high-dose intravenous melphalan and dexamethasone followed by autologous stem cell transplantation [3, 4]. Liver transplantation may be considered for selected patients with advanced hepatic amyloidosis. Liver transplant has been successful in patients with familial forms of amyloidosis and in a few cases of AL amyloidosis [8]. However, in AL amyloidosis, liver transplantation is not curative, and these patients require chemotherapy due to the high rate of recurrence [8]. Other treatment options focus on supportive and symptomatic relief. In patients presenting with recurrent ascites, dietary modifications, diuretics, and paracentesis may help alleviate discomfort associated with abdominal distension. Patients who have undergone prior stem cell transplant may be at risk for developing sinusoidal obstruction syndrome which can also cause ascites but is associated with other findings of liver biopsy.

To conclude, this case highlights the importance of considering hepatic amyloidosis in the differential diagnosis when a patient presents with unexplained recurrent ascites to avoid delays in diagnosis and subsequent treatment. Amyloid deposition occurs either within the sinuses or the liver parenchyma itself to cause this rare disease. The diagnosis of hepatic amyloidosis is difficult as patients typically present with nonspecific symptoms such as fatigue, hepatomegaly, or an elevated alkaline phosphatase and imaging is nonspecific as well. Definitive diagnosis occurs with liver biopsy and subsequent pathological analysis with Congo red staining. The treatment options are limited and aimed at symptom management. While a liver transplant may help patients with familial amyloidosis, it is not often a good option for patients with AL amyloidosis as long-term outcomes are poor. Most patients have a limited prognosis and a high one-year mortality.

## Data Availability

Data sharing is not applicable to this article as no datasets were generated or analyzed during the current study.
